# Effects of BPA in Snails: Oehlmann et al. Respond

**Published:** 2006-06

**Authors:** Jörg Oehlmann, Ulrike Schulte-Oehlmann, Matthias Oetken, Jean Bachmann, Ilka Lutz, Werner Kloas, Thomas A. Ternes

**Affiliations:** Johann Wolfgang Goethe University, Frankfurt am Main, Institute of Ecology, Evolution and Diversity, Frankfurt am Main, Germany, E-mail: oehlmann@zoology.uni-frankfurt.de; Federal Environmental Agency, Section Ecological Assessment of Substances, Dessau, Germany; Leibniz-Institute of Freshwater Ecology and Inland Fisheries, Department of Inland Fisheries, Berlin, Germany; Federal Institute of Hydrology, Koblenz, Germany

We welcome critical appraisals that help to provide balance; however, Dietrich et al. gave an unjustified reproach. We feel that Dietrich’s position is severely compromised because he serves as an expert for the bisphenol A (BPA) Industry Group (Brussels, Belgium). We would like to respond to the issues raised by Dietrich et al., as well as to their oversights and inappropriate interpretations of our findings.

The source of test animals was clearly provided in our “Materials and Methods” (Oehlmann et al. 2006). All animals were dissected and sexed; thus, sex distribution was known for each time-point of the experiment. We supposed a 1:1 sex ratio for dead snails, although historical data (*n* > 14,000) indicate a slight prevalence of females (1.13:1); therefore, our assumption was conservative. Egg production was corrected for the number of females in the tanks, and snail densities were equal for all groups at each time-point.

Semistatic designs are widely applied in scientific and regulatory ecotoxicology [Organization for Economic Development and Co-operation (OECD) 1998]. The actual exposure concentrations of BPA were measured and clearly communicated in our Tables 1 and 2 (Oehlmann et al. 2006). Because 17α-ethinylestradiol (EE_2_) is more stable than BPA (Larsson et al. 1999), exposure to the positive control is also guaranteed in our 24-hr renewal test. Interestingly, Dietrich himself coauthored a semistatic study on snails (Czech et al. 2001) with several shortcomings: they used no analytical verification of exposure concentrations, no replicates, and inconsistent group size.

Analysis of covariance (ANCOVA) analyses of fecundity, development, and other cumulative data are widely used (Bochdansky and Bollens 2004; Dziminski and Alford 2005; Schärer and Wedekind 1999). In our experiment 2 with replicates (Oehlmann et al. 2006), ANOVA confirmed the ANCOVA results (Figure 2A,2C). A BPA Industry Group–sponsored statistical reevaluation of our raw data (Ecostat 2005) concluded that “at 20°C the mean egg production increased compared to the control in the BPA-exposed females at all applied concentrations (0.25, 0.5, 1 and 5 μg/L), and decreased in the BPA+faslodex- or tamoxifen-exposed females.”

We achieved an association for a steady state of specific binding in three independent time-course studies (Oehlmann et al. 2006). We determined nonspecific binding using a 1,000-fold excess of unlabeled ligands resulting in clear specific binding for testosterone and estradiol. At higher concentrations, nonspecific binding was 70%, comparable with findings of Chou and Dietrich (1999), who also performed their experiments in duplicate. This percentage might be due to homogenization of large amounts of tissue with high protein content but a limited degree of specific cytosolic binding sites. In our study (Oehlmann et al. 2006), we did not intend to deliver a complete binding study in which saturation experiments with Scatchard analysis are needed, but to provide indications for the presence of estrogen receptors by a specific binding of ligands to cytosolic extracts (a widely used practice). Tamoxifen was not disqualified as an antiestrogen because it elicited a binding higher than that of BPA. Furthermore, *in vitro* ligand affinities have a limited predictive value for biologic potencies *in viv*o (Kloas et al. 1999). In summary, the binding study was performed appropriately for the desired purpose and provides initial evidence for specific estrogen binding sites with high affinity for BPA.

Data presented in our Figure 1B (Oehlmann et al. 2006) were published in Schulte-Oehlmann et al. (2001) without EE_2_ because the focus of that work was comparing responses to BPA in four prosobranch species, including *Marisa*. Because the article was published in German, the distribution was not large enough to bring the issue to a wider audience. In the current article (Oehlmann et al. 2006), EE_2_ data were included to demonstrate the masking of BPA effects during the spawning season. Because future BPA industry-sponsored studies intend to investigate BPA effects under conditions maximizing reproduction, the problem of masked effects and an associated loss of sensitivity is of vital importance.

Responses in *Marisa* (ruptured oviducts, increased spawning) are estrogen specific and opposite of androgenic effects (imposex, reduced spawning). This and evidence communicated in our article (Oehlmann et al. 2006) justify the use of EE_2_ to demonstrate the responsiveness of organisms. Non-monotonic concentration responses have also been reported for estrogen-regulated end points in EE_2_-exposed fish (Pawlowski et al. 2004), supporting our view that estrogen-specific binding sites in *Marisa* may represent functional receptors.

Dietrich et al.’s charges that our “Introduction” and “Discussion” were “imbalanced and indeed alarmist” and that we selectively used literature are unjustified.

We hope that the evidence presented here serves to refute the unjustified claims made by Dietrich et al. We leave it to the readers to make final judgment, but we feel that with the ever-increasing body of evidence showing effects of BPA on reproduction in various animal species, common sense will eventually prevail on this issue.
